# RCAS1 as a tumour progression marker: an independent negative prognostic factor in gallbladder cancer

**DOI:** 10.1054/bjoc.2001.2192

**Published:** 2001-12

**Authors:** T Oshikiri, Y Hida, M Miyamoto, H Hashida, K Katoh, M Suzuoki, Y Nakakubo, K Hiraoka, T Shinohara, T Itoh, S Kondo, H Katoh

**Affiliations:** Department of Surgical Oncology, Division of Cancer Medicine, Hokkaido University Graduate School of Medicine, North 15, West 7, Kita-ku, Sapporo, 060-8638, Japan; Department of Pathology, Teinekeijinkai Hospital, 1–12, Maeda, Teine-ku, Sapporo, 006–0811, Japan; Department of Surgical Pathology, Hokkaido University Hospital, North 15, West 7, Kita-ku, Sapporo, 060-8638, Japan

**Keywords:** RCAS1, gallbladder cancer, carcinogenesis, immunohistochemistry

## Abstract

Receptor-binding cancer antigen expressed on SiSo cells (RCAS1) induces apoptosis in immune cells bearing the RCAS1 receptor. We sought to determine RCAS1 involvement in the origin and progression of gallbladder cancer, and also implications of RCAS1 for patient survival. RCAS1 expression was examined immunohistochemically in 110 surgically resected gallbladder specimens. The gallbladders represented 20 cases of cholecystitis with no associated pancreaticobiliary maljunction; 23 cases of cholecystitis with pancreaticobiliary maljunction; 14 cases of adenomyomatosis; 7 adenomas; and 46 cancers. High expression of RCAS1 (immunoreactivity in over 25% of cells) was observed in 32 of the 46 cancers (70%), but not in other diseases, including pre-cancerous conditions. RCAS1 immunoreactivity was associated with depth of tumour invasion (*P* = 0.0180), lymph node metastasis (*P* = 0.0033), lymphatic involvement (*P* = 0.0104), venous involvement (*P* = 0.0224), perineural involvement (*P* = 0.0351) and stage by the tumour, nodes and metastases (TNM) classification (*P* = 0.0026). Thus, RCAS1 expression may be a relatively late event in gallbladder carcinogenesis ** possibly promoting tumour progression. Cox regression multivariate analysis demonstrated RCAS1 positivity to be an independent negative predictor for survival (*P* = 0.0337; risk ratio, 12.690; 95% confidence interval, 1.216–132.423). High expression of RCAS1 significantly correlated with tumour progression and predicted poor outcome in gallbladder cancer. © 2001 Cancer Research Campaign http://www.bjcancer.com


*British Journal of Cancer* (2001) **85**(12), 1922–1927
© 2001 Cancer Research Campaign
doi: 10.1054/ bjoc.2001.2192, available online at http://www.idealibrary.com on IDEAL

### RCAS1 as a tumour progression marker: an independent negative prognostic factor in gallbladder cancer

**T Oshikiri[1], Y Hida[1], M Miyamoto[1], H Hashida[1], K Katoh[1], M Suzuoki[1], Y Nakakubo[1], K Hiraoka[1], T Shinohara[2], T Itoh[3], S Kondo[1] and H Katoh[1]**

[1]Department of Surgical Oncology, Division of Cancer Medicine, Hokkaido University Graduate School of Medicine, North 15, West 7, Kita-ku, Sapporo, 060-8638, Japan; [2]Department of Pathology, Teinekeijinkai Hospital, 1–12, Maeda, Teine-ku, Sapporo, 006–0811, Japan; [3]Department of Surgical Pathology, Hokkaido University Hospital, North 15, West 7, Kita-ku, Sapporo, 060-8638, Japan

**Summary** Receptor-binding cancer antigen expressed on SiSo cells (RCAS1) induces apoptosis in immune cells bearing the RCAS1 receptor. We sought to determine RCAS1 involvement in the origin and progression of gallbladder cancer, and also implications of RCAS1 for patient survival. RCAS1 expression was examined immunohistochemically in 110 surgically resected gallbladder specimens. The gallbladders represented 20 cases of cholecystitis with no associated pancreaticobiliary maljunction; 23 cases of cholecystitis with pancreaticobiliary maljunction; 14 cases of adenomyomatosis; 7 adenomas; and 46 cancers. High expression of RCAS1 (immunoreactivity in over 25% of cells) was observed in 32 of the 46 cancers (70%), but not in other diseases, including pre-cancerous conditions. RCAS1 immunoreactivity was associated with depth of tumour invasion ($P$ = 0.0180), lymph node metastasis ($P$ = 0.0033), lymphatic involvement ($P$ = 0.0104), venous involvement ($P$ = 0.0224), perineural involvement ($P$ = 0.0351) and stage by the tumour, nodes and metastases (TNM) classification ($P$ = 0.0026). Thus, RCAS1 expression may be a relatively late event in gallbladder carcinogenesis, possibly promoting tumour progression. Cox regression multivariate analysis demonstrated RCAS1 positivity to be an independent negative predictor for survival ($P$ = 0.0337; risk ratio, 12.690; 95% confidence interval, 1.216–132.423). High expression of RCAS1 significantly correlated with tumour progression and predicted poor outcome in gallbladder cancer. © 2001 Cancer Research Campaign http://www.bjcancer.com

**Keywords**: RCAS1; gallbladder cancer; carcinogenesis; immunohistochemistry

Gallbladder cancer (GBC) has an extremely poor prognosis. Resection for cure has been reported to be possible in only 20–40% of cases, and 5-year survival rates following such resection have ranged from 5–40% (Ogura et al, 1991; Bartlett et al, 1996; Benoist et al, 1998). Recent advances in hepatobiliary imaging, introduction of extended operations combining liver resection with wide lymph node dissection, and increases in incidental histologic diagnosis of early-stage cancer following laparoscopic cholecystectomy are beginning to improve the prognosis of GBC (Bartlett et al, 1996; Shimada et al, 1997). As another result, new controversies have emerged in the surgical management of GBC, especially concerning the incidentally discovered cases. Specifically, questions persist as to whether reoperation to achieve wider resection is indicated when tumours are discovered incidentally at histologic examination after simple cholecystectomy. More complete information for planning therapeutic strategies and for developing therapy is needed to further improve clinical outcome. This need includes a better understanding of the molecular basis of gallbladder carcinogenesis and identification of prognostically important biologic markers.

A mouse monoclonal antibody designated 22-1-1 was produced against a human uterine adenocarcinoma cell line, SiSo, in 1996 (Sonoda et al, 1995; Sonoda et al, 1996). A cDNA encoding the antigen recognized by the 22-1-1 antibody was termed ‘receptor binding cancer antigen expressed on SiSo cells’, or RCAS1. Cancers escape immune surveillance by a variety of mechanisms, including some evasive strategies to avoid immune recognition, but also active modulation and suppression of immune cell function. The RCAS1 gene product is a membrane constituent that acts as a ligand for a putative receptor present on various human cells including normal peripheral lymphocytes such as T, B, and natural killer cells. RCAS1 inhibited growth of receptor-expressing cells in vitro and induced apoptotic cell death. Given these results, tumour cells could evade immune surveillance by expression of RCAS1, which would act to suppress clonal expansion and induce apoptosis in immune cells possessing RCAS1 receptors (Nakashima et al, 1999).

In uterine, ovarian and lung cancer, RCAS1 expression has been associated with a poorer prognosis in those cancers (Sonoda et al, 1996; Kaku et al, 1999; Iwasaki et al, 2000). Whether expression of this antigen is related to carcinogenesis, tumour progression, and prognosis in other organs is not known.

To elucidate the role of RCAS1 in carcinogenesis and tumour progression in GBC, we immunohistochemically investigated RCAS1 expression in 110 surgically resected gallbladder specimens, including pre-cancerous as well as cancerous lesions. In case of cancer, we analysed the relationship between RCAS1 immunopositivity and clinical and histopathologic features.

*Received 31 May 2001*
*Revised 30 August 2001*
*Accepted 20 September 2001*

*Correspondence to:* T Oshikiri

### MATERIAL AND METHODS

### Patients and tissue specimens

Between 1989 and 1999, a total of 110 gallbladders were resected in the Second Department of Surgery at the Hokkaido University School of Medicine and the Departments of Surgery at Hokkaido Gastroenterology Hospital and Teinekeijinkai Hospital. Gallbladder specimens included seven adenomas, 14 cases of adenomyomatosis, 23 cases of cholecystitis associated with pancreaticobiliary maljunction (PBM), 20 cases of cholecystitis without PBM, and 46 carcinomas. Among the seven gallbladder adenomas six were tubular and 1 was papillary. The ages of patients with cancer ranged from 32–86 years (median 67); 17 patients were men and 29 were women. Patients were grouped according to age as being either < or ≥ 67 years old. The clinicopathologic features of these cases were used to evaluate the lesion according to the pathologic tumour node and metastases (pTNM) classification of the International Union Against Cancer (Sobin et al, 1997). Among all 46 patients, 42 had systematic follow-up examinations. The median duration of follow-up was 41.0 months (range 3.1–40.8 months).

### Immunohistochemistry

Surgical specimens were fixed in 10% formalin solution and embedded by routine methods in paraffin for sectioning at a thickness of 4 µm. Sections were then deparaffinized in xylene and rehydrated through a graded ethanol series. Endogenous peroxidase was blocked with 3% hydrogen peroxide for 10 min. Sections then were washed twice in phosphate-buffered saline (PBS) and incubated with 10% normal goat serum (Histofine SAB-PO kit; Nichirei, Tokyo, Japan) for 30 min. Primary antibody (anti-RCAS1 mouse monoclonal antibody, Medical & Biological Laboratories, Tokyo, Japan) was applied in a 1:500 dilution in PBS, and the sections were incubated overnight at 4˚C. After 3 additional washes, sections were incubated with polyvalent biotinylated goat anti-mouse antibody for 30 min at room temperature. Sections were washed 3 times in PBS and incubated with streptavidin-conjugated peroxidase for 30 min. After 3 additional washes, reaction product was visualized by incubation with 3,3′-diaminobenzidine tetrahydrochloride (Histofine SAB-PO kit; Nichirei Tokyo, Japan) for approximately 15 min, followed by washing in distilled water. Sections then were counterstained in haematoxylin for 1 min and mounted in Permount. As a positive control, sections of a uterine adenocarcinoma previously found to express the RCAS1 protein were affixed to the same slides as the gallbladder sections and were stained in parallel. In a negative control, non-immune purified mouse IgM was substituted for the primary antibody.

### Evaluation of immunoreactivity

The degree of RCAS1 immunoreactivity was used to classify cases into four categories according to widely used criteria (Loda et al, 1997; Porter et al, 1997; Anayama et al, 1998). In RCAS1 I cases, fewer than 25% of tumour cells showed immunoreactivity; in RCAS1 II, 25–50%; in RCAS1 III, 50–75%; and in RCAS1 IV, more than 75%. RCAS1 II, III, and IV were considered high-expression patterns, while RCAS1 I was considered to be low expression. Scoring of RCAS1 immunoreactivity was based on examination of 10 high-power (× 400) microscopic fields equivalent to 1000 cells for each section. Immunoreactivity in each section was reviewed by each of three investigators. In cases of occasional discrepancy in the interpretation, consensus was achieved after discussion with the aid of a multiheaded microscope.

### Statistical analysis

The $\chi^2$ test or Fisher's test was used as appropriate to evaluate the significance of RCAS1 status with respect to various clinicopathologic parameters. The Kaplan–Meier method was used to estimate overall survival. Survival differences according to RCAS1 expression were analyzed by the log rank test. The influence of variables on survival was assessed using Cox univariate and multivariate regression analyses. Probability values of less than 0.05 were regarded as indicating significance.

### RESULTS

### RCAS1 expression in cases of cholecystitis, adenomyomatosis and adenoma

RCAS1 immunoreactivity was present in less than 5% of cells in all 20 cases of cholecystitis without PBM (Figure 1A), all 14 cases of adenomyomatosis (Figure 1B), all 23 cases of cholecystitis with PBM (Figure 1C), and in all 7 cases of adenomas (Figure 1D).

### RCAS1 expression in GBC

A total of 46 GBCs were grouped as 14 RCAS1 I tumours (30%; Figure 1E); 7 RCAS1 II tumours (15%; Figure 1F); 10 RCAS1 III tumours (22%; Figure 1G); and 15 RCAS1 IV tumours (33%; Figure 1H). Thus, 32 tumours (70%) were classified as showing high RCAS1 expression (more than 25% of cells).

The frequency of high RCAS1 expression increased with tumour stage according to the pTNM system: 44.4% of stage I (4 of a total of 9 cases), 53.3% of stage II (8 of a total of 15 cases), 85.7% of stage III (6 of a total of 7 cases), and 93.3% of stage IV (14 of a total of 15 cases; Table 1). RCAS1 immunoreactivity showed a significant relationship to depth of tumour invasion ($P = 0.0180$), lymph node metastasis ($P = 0.0033$), lymphatic involvement ($P = 0.0104$), venous involvement ($P = 0.0224$), perineural involvement ($P = 0.0351$), and pTNM stage ($P = 0.0026$) by the $\chi^2$ test. No significant association was noted between RCAS1 expression and other clinicopathologic features (Table 2).

### Kaplan–Meier survival analysis

Survival curves constructed according to the Kaplan–Meier method are shown in Figures 2 and 3. In 42 patients with GBC, survival in patients with a high RCAS1 expression pattern was

**Table 1** RCAS1 expression in GBCs by tumour stage

| Histopathologic stage[a] | No. of cases | No. of positive cases |
|---|---|---|
| I | 9 | 4 (44.4%) |
| II | 15 | 8 (53.3%) |
| III | 7 | 6 (85.7%) |
| IV | 15 | 14 (93.3%) |

[a]Staging was carried out by the pTNM system.

© 2001 Cancer Research Campaign

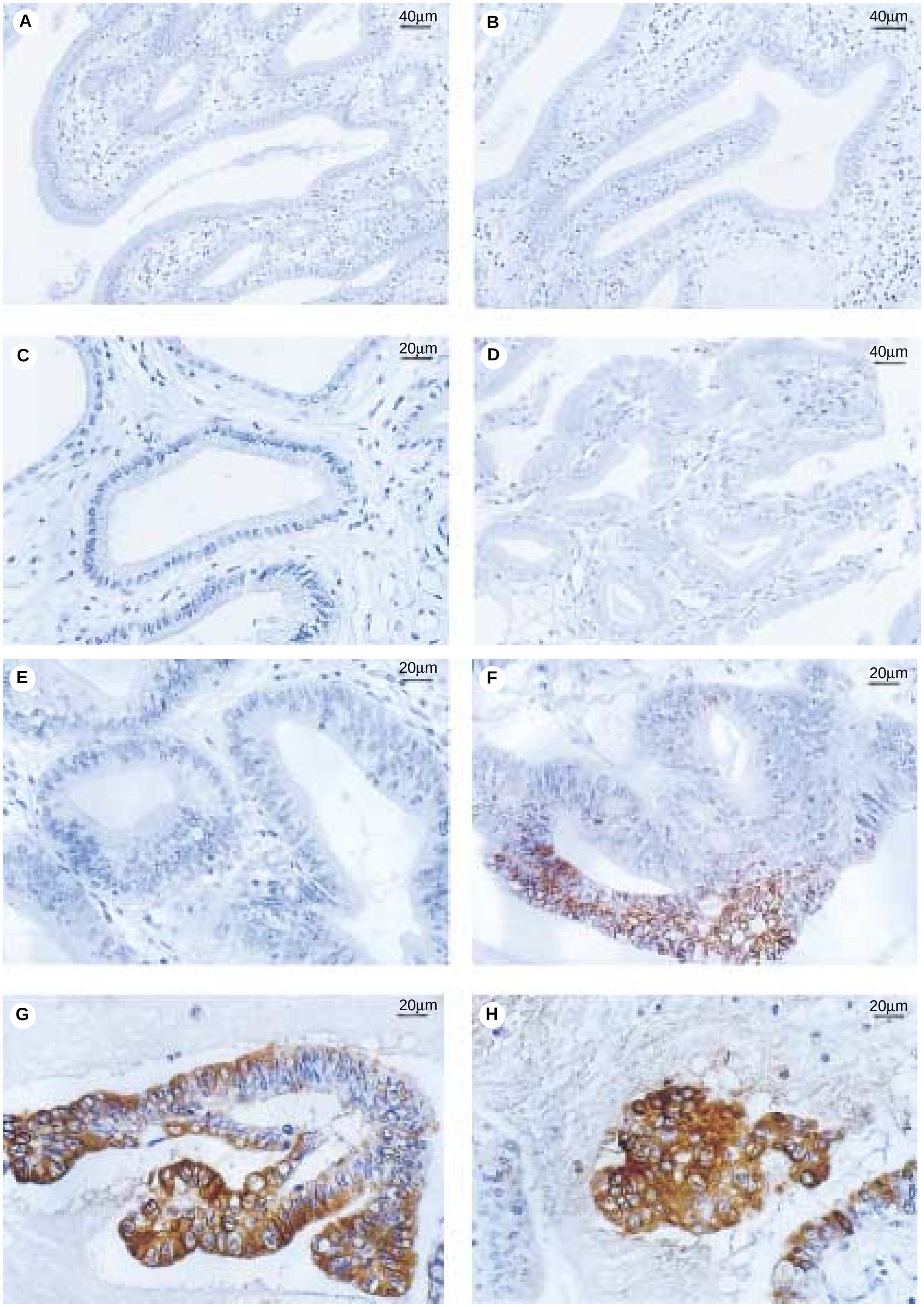

**Figure 1** Representative examples of RCAS1 immunohistochemistry. RCAS1 immunoreactivity was observed in less than 5% of cells of (**A**) cases of cholecystitis without pancreaticobiliary maljunction (PBM), (**B**) cholecystitis with PBM, (**C**) adenomyomatosis, and (**D**) adenoma. In cancer cells, RCAS1 was demonstrated both in the cytoplasm and on the cell membrane. Cancers were classified as (**E**) RCAS1 I (less than 25% of cells stained), (**F**) RCAS1 II (25–50% of cells stained), (**G**) RCAS1 III (50–75% of cells stained), or (**H**) RCAS1 IV (more than 75% of cells stained). (Original magnification (**A**, **B**, **D**) × 200, (**C**, **E**, **F**, **G**, **H**] × 400)

 *© 2001 Cancer Research Campaign*

**Table 2** Association of RCAS1 expression with selected clinicopathologic characteristics in patients with GBC

| Variable | RCAS1 expression | | *P*-value[a] |
|---|---|---|---|
| | Positive (%) | Negative (%) | |
| Patients (*n*) | 32 (70) | 14 (30) | |
| Age (years) | | | 0.2767 |
| < 67 | 17 (77) | 5 (23) | |
| ≥ 67 | 15 (63) | 9 (37) | |
| Sex | | | 0.2254 |
| Male | 10 (59) | 7 (41) | |
| Female | 22 (76) | 7 (24) | |
| Histologic type | | | 0.1037 |
| Papillary/well | 17 (61) | 11 (39) | |
| Moderately/poorly | 15 (83) | 3 (17) | |
| Depth of tumour invasion | | | 0.0180 |
| Tis, $T_1$, $T_2$ | 18 (58) | 13 (42) | |
| $T_3$, $T_4$ | 14 (93) | 1 ( 7) | |
| Lymph node metastasis | | | 0.0033 |
| Negative | 15 (54) | 13 (46) | |
| Positive | 17 (94) | 1 ( 6) | |
| Lymphatic involvement | | | 0.0104 |
| Negative | 12 (52) | 11 (48) | |
| Positive | 20 (87) | 3 (13) | |
| Venous involvement | | | 0.0224 |
| Negative | 16 (57) | 12 (43) | |
| Positive | 16 (89) | 2 (11) | |
| Perineural involvement | | | 0.0351 |
| Negative | 17 (59) | 12 (41) | |
| Positive | 15 (88) | 2 (12) | |
| Stage by TNM[b] | | | 0.0026 |
| Stage I or II | 12 (50) | 12 (50) | |
| Stage III or IV | 20 (91) | 2 ( 9) | |

[a]From the $\chi^2$ test. [b]TNM: tumour, nodes and metastases; is: in situ.

significantly worse than in those with a low-expression pattern (log rank test, $P = 0.0001$; Figure 2). A similar tendency was noted in 21 patients with pT2 cancer (log rank test, $P = 0.0074$; Figure 3).

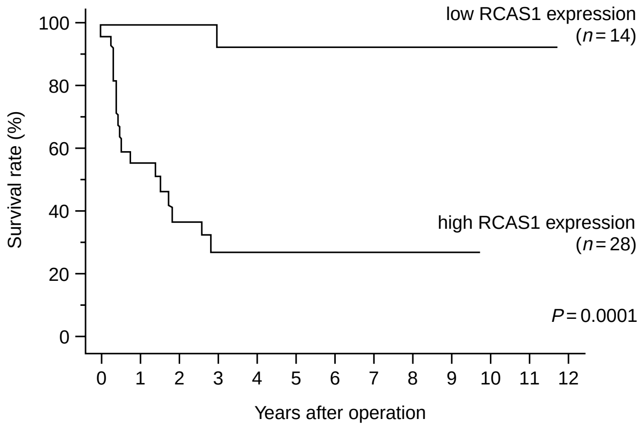

**Figure 2** Kaplan–Meier curves for overall survival according to RCAS1 expression (high expression vs low expression, compared using the log rank test) in 42 patients who underwent radical surgery for gallbladder cancer

### Univariate survival analysis

Univariate analysis performed by Cox regression identified age ($P = 0.0064$), histologic tumour type ($P = 0.0036$), depth of tumour invasion ($P < 0.0001$), lymph node metastasis ($P < 0.0001$), lymphatic involvement ($P = 0.0010$), venous involvement ($P = 0.0001$), perineural involvement ($P = 0.0003$), and RCAS1 positivity ($P = 0.0047$) as showing significant correlation with survival in 42 patients with GBC (Table 3). For 21 patients with pT2 cancer, only RCAS1 positivity ($P = 0.0257$) was associated with a significant difference in patient survival (Table 4).

### Multivariate survival analysis

Factors that showed a significant difference in patient survival by univariate survival analysis were considered further in a multivariate survival analysis. Cox regression multivariate analysis indicated that depth of tumour invasion ($P = 0.0175$), lymph node

**Table 3** Evaluation of possible prognostic factors including RCAS1 expression in 42 patients with GBC using Cox regression analysis

| Variables | Univariate analysis | | | Multivariate analysis | | |
|---|---|---|---|---|---|---|
| | Risk ratio | 95% CI | *P*-value | Risk ratio | 95% CI | *P*-value |
| Age (≥ 67/< 67) | 3.874 | 1.462–10.263 | 0.0064 | 2.117 | 0.576–7.789 | 0.2589 |
| Sex (F/M) | 1.299 | 0.526–3.207 | 0.5699 | | | |
| Histologic type<br>Pap and well/mod and poor) | 4.074 | 1.582–10.489 | 0.0036 | 0.266 | 0.030–2.364 | 0.2348 |
| Depth of tumor invasion<br>(Tis, $T_1$, $T_2$/$T_3$, $T_4$) | 12.230 | 4.390–34.072 | < 0.0001 | 13.179 | 1.571–110.575 | 0.0175 |
| Lymph node metastasis<br>(Negative/positive) | 11.019 | 4.032–30.114 | < 0.0001 | 4.918 | 1.127–21.459 | 0.0341 |
| Lymphatic involvement<br>(Negative/positive) | 5.195 | 1.940–13.991 | 0.0010 | 4.048 | 0.931–17.592 | 0.0621 |
| Venous involvement<br>(Negative/positive) | 6.726 | 2.564–17.644 | 0.0001 | 1.784 | 0.194–16.371 | 0.6088 |
| Peri-neural involvement<br>(Negative/positive) | 5.526 | 2.173–14.056 | 0.0003 | 0.418 | 0.056–3.102 | 0.3948 |
| RCAS1 (–/+) | 18.763 | 2.456–143.367 | 0.0047 | 12.690 | 1.216–132.423 | 0.0337 |

Pap: papillary adenocarcinoma; well: well-differentiated adenocarcinoma; Mod: moderately differentiated adenocarcinoma; poor: poorly differentiated adenocarcinoma; is: in situ.

© 2001 Cancer Research Campaign

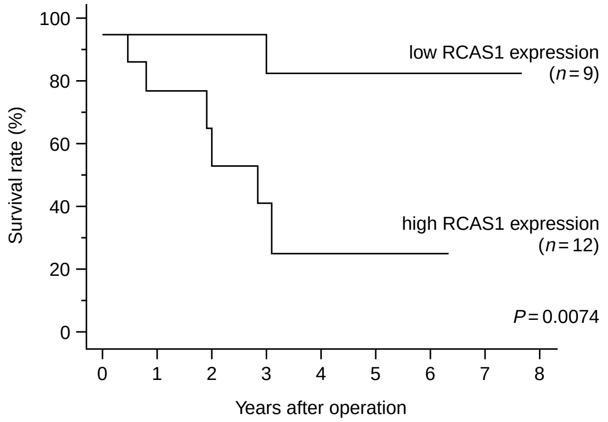

**Figure 3** Kaplan–Meier curves for overall survival according to RCAS1 expression (high expression vs low expression, compared using the log rank test) in 21 patients with pT2 gallbladder cancer

**Table 4** Prognostic value of RCAS1 expression and other variables in 21 patients with pT2 GBC using Cox regression analysis

| Variables | Univariate analysis | | |
|---|---|---|---|
| | Risk ratio | 95% CI | *P*-value |
| Age (≥ 67/< 67) | 3.269 | 0.724–14.750 | 0.1234 |
| Sex (F/M) | 1.723 | 0.383–7.750 | 0.4781 |
| Histologic type (Pap and well/mod and poor) | 1.950 | 0.431–8.832 | 0.3861 |
| Lymph node metastasis (Negative/positive) | 4.287 | 0.951–19.320 | 0.0580 |
| Lymphatic involvement (Negative/positive) | 2.822 | 0.614–12.969 | 0.1824 |
| Venous involvement (Negative/positive) | 1.612 | 0.311–8.349 | 0.5691 |
| Peri-neural involvement (Negative/positive) | 0.684 | 0.082–5.696 | 0.7252 |
| RCAS1 (–/+) | 12.560 | 1.360–115.990 | 0.0257 |

Pap: papillary adenocarcinoma; well: well-differentiated adenocarcinoma; Mod: moderately differentiated adenocarcinoma; poor: poorly differentiated adenocarcinoma; is: in situ.

metastasis (*P* = 0.0341), and RCAS1 positivity (*P* = 0.0337) were independent unfavourable factors in 42 patients with GBC (Table 3).

### DISCUSSION

The present study showed that:

1. Occurrence of high RCAS1 expression increases as tumour stage advances
2. High RCAS1 expression is related to depth of tumour invasion, lymph node metastasis, and invasion of lymphatics, veins, and perineural areas
3. High RCAS1 expression is an independent unfavourable prognostic factor.

We investigated various gallbladder lesions, some considered pre-cancerous, in order to evaluate possible involvement of RCAS1 in carcinogenesis. Conditions examined included PBM, adenomyomatosis, and adenoma. The incidence of biliary cancer in patients with PBM has been reported as 23.6–37.3% (Aoki et al, 1987; Uchimura et al, 1991). On the other hand, adenomyomatosis of the gallbladder is generally considered to carry no risk of malignant transformation, although a few cases have been reported where adenocarcinoma was associated with adenomyomatosis (Katoh et al, 1988; Ootani et al, 1992). Adenoma has been characterized as a precursor of GBC (Kozuka et al, 1982). We immunostained sections of pre-cancerous lesions together with sections from positive control specimens affixed to the same glass slides to minimize the chance of a false-negative result. In these cases, only the control section showed strong RCAS1 immunoreactivity. Cancer-specific genes that have been relatively well investigated in GBC include p53 and K-ras. Mutations at these loci are considered to be early events in gallbladder carcinogenesis, being likely to participate in transforming pre-cancerous lesions to cancer but unlikely to influence the clinical properties of GBC (Ajiki et al, 1996a, 1996b). In our immunohistochemical study, high expression of RCAS1 was observed frequently in GBC (70%), but was never seen in pre-cancerous lesions. This suggests that RCAS1 is probably not involved in the origins of GBC. Instead, RCAS1 appears to be a late event, which means tumour progression and not malignant transformation, as a part of a multistep process of gallbladder carcinogenesis.

Strong expression of RCAS1 by GBC was significantly associated with depth of tumour invasion, lymph node metastasis, lymphatic involvement, venous involvement, and peri-neural involvement. An association between RCAS1 expression and depth of tumour invasion has been reported for non-small cell lung cancer (Iwasaki et al, 2000). Iwasaki et al reported more frequent occurrence of apoptosis in tumour-infiltrating lymphocytes in lung cancers strongly expressing RCAS1 than in lung cancers showing less expression. One possible explanation for those findings is that GBC cells expressing RCAS1 may escape immune surveillance and survive in lymphatic vessels, lymph nodes, and the extracellular matrix. In addition, RCAS1-expressing cells may have a survival advantage over tumour cells not expressing the protein, so cells expressing RCAS1 would constitute an increasing fraction of tumour cells as invasion deepened. As a tumour penetrates more deeply, it is more likely to make contact with lymphatic, venous, and peri-neural structures in the submucosal layer. Expression of RCAS1, then, would be expected to correlate with involvement of these structures.

For patients who have undergone cholecystectomy with a subsequent incidental finding of gallbladder cancer in the resected specimen, radical re-operation may be necessary if the tumour has extended into peri-muscular connective tissue (T2) and expresses RCAS1. In particular, a patient whose tumour shows high RCAS1 expression may be a candidate for extended resection including lymph node dissection, since lymph node metastasis is likely.

A particularly important finding is that RCAS1 expression was associated with a short period of survival in patients who have had a resectable GBC. Cox regression univariate analysis showed high expression of RCAS1 to be the prognostic factor among pT2 cancers. Surgical resection failed to cure the patients with GBC showing high expression of RCAS1 protein, and advanced cancer with such expression requires aggressive therapy. As new therapeutic strategies, targeting of tumours using antibodies against cancer specific antigens may be candidate. Several studies of radioimmunotherapy using radiolabelled anti-carcinoembryonic antigen (CEA) or anti-CD22 have been reported (Casey et al, 1999; Linden et al, 1999). Our data suggested that RCAS1 could be a potential target for GBC radioimmunotherapy.

 *© 2001 Cancer Research Campaign*

In conclusion, we have found that RCAS1 participates in progression of GBC and that strong immunoreactivity for RCAS1 predicts a poor outcome.

### REFERENCES

Ajiki T, Fujimori T, Onoyama H, Yamamoto M, Kitazawa S, Maeda S and Saitoh Y (1996a) K-ras gene mutation in gall bladder carcinomas and dysplasia. *Gut* **38**: 426–429

Ajiki T, Onoyama H, Yamamoto M, Asaka K, Fujimori T, Maeda S and Saitoh Y (1996b) p53 protein expression and prognosis in gall bladder carcinoma and premalignant lesions. *Hepatogastroenterology* **43**: 521–526

Anayama T, Furihara M, Ishikawa T, Ohtsuki Y and Ogoshi S (1998) Positive correlation between $p27^{Kipl}$ expression and progression of human esophageal squamous cell carcinoma. *Int J Cancer* **79**: 439–443

Aoki H, Sugatani H and Shimazu M (1987) A clinical study on cancer of the bile duct associated with anomalous arrangement of pancreaticobiliary ductal system: analysis of 569 cases collected in Japan. *J Biliary Tract Pancreas* **8**: 1539–1551

Bartlett DL, Fong Y, Fortner JG, Brennan MF and Blumgart LH (1996) Long-term results after resection for gallbladder cancer: implications for staging and management. Review. *Ann Surg* **224**: 639–646

Benoist S, Panis Y and Fagniez PL (1998) Long-term results after curative resection for carcinoma of the gallbladder: French University Association for Surgical Research. *Am J Surg* **175**: 118–122

Casey JL, Pedley RB, King DJ, Green AJ, Yarranton GT and Begent RH (1999) Dosimetric evaluation and radioimmunotherapy of anti-tumour multivalent Fab' fragments. *Br J Cancer* **81**: 972–980

Iwasaki T, Nakashima M, Watanabe T, Yamamoto S, Inoue Y, Yamanaka H, Matsumura A, Aluchi K, Mori T and Okada M (2000) Expression and prognostic significance in lung cancer of human tumor-associated antigen RCAS1. *Int J Cancer* **89**: 488–493

Kaku T, Sonoda K, Kamura T, Hirakawa T, Sakai K, Amada S, Ogawa S, Kobayashi H, Nakashima M, Watanabe T and Nakano H (1999) The prognostic significance of tumor-associated antigen 22-1-1 expression in adenocarcinoma of the uterine cervix. *Clin Cancer Res* **5**: 1449–1453

Katoh T, Nakai T, Hayashi S and Satake T (1988) Noninvasive carcinoma of the gallbladder arising in localized type adenomyomatosis. *Am J Gastroenterol* **83**: 670–674

Kozuka S, Tsubone M, Yasui A and Hachisuka K (1982) Relation of adenoma to carcinoma in the gallbladder. *Cancer* **50**: 2226–2234

Linden O, Tennvall J, Cavallin-Stahl E, Darte L, Garkavij M, Lindner KJ, Ljungberg M, Ohlsson T, Sjogreen K, Wingardh K and Strand SE (1999) Radioimmunotherapy using 131I-labeled anti-CD22 monoclonal antibody (LL2) in patients with previously treated B-cell lymphomas. *Clin Cancer Res* **5**: 3287s–3291s

Loda M, Cukor B, Tam SW, Lavin P, Piorentino M, Draetta GF, Jessup JM and Pagano M (1997) Increased proteasome-dependent degradation of the cyclin-dependent kinase inhibitor p27 in aggressive colorectal carcinomas. *Nat Med* **3**: 231–234

Nakashima M, Sonoda K and Watanabe T (1999) Inhibition of cell growth and induction of apoptotic cell death by the human tumor-associated antigen RCAS1. *Nat Med* **5**: 938–942

Ogura Y, Mizumoto R, Isaji S, Kusuda T, Matsuda S and Tabata M (1991) Radical operations for carcinoma of the gallbladder: present status in Japan. *World J Surg* **15**: 337–343

Ootani T, Shirai Y, Tsukada K and Muto T (1992) Relationship between gallbladder carcinoma and the segmental type of adenomyomatosis of the gallbladder. *Cancer* **69**: 2647–2653

Porter PL, Malone KE, Heagerty PJ, Alexander GM, Gatti LA, Firpo EJ, Daling JR and Roberts JM (1997) Expression of cell-cycle regulators $p27^{Kipl}$ and cyclin E, alone and in combination, correlate with survival in young breast cancer patients. *Nat Med* **3**: 222–225

Shimada H, Endo I, Togo S, Nakano A, Izumi T and Nakagawara G (1997) The role of lymph node dissection in the treatment of gallbladder carcinoma. *Cancer* **79**: 892–899

Sobin LH and Wittekind CH (1997) TNM classification of malignant tumours. 5th edn, Wiley-Liss: New York

Sonoda K, Nakashima M, Saito T, Amada S, Kamura T, Nakano H and Watanabe T (1995) Establishment of a new human uterine cervical adenocarcinoma cell line, SiSo, and its reactivity to anticancer reagents. *Int J Oncol* **6**: 1099–1194

Sonoda K, Nakashima M, Kaku T, Kamura T, Nakano H and Watanabe T (1996) A novel tumor-associated antigen expressed in human uterine and ovarian carcinomas. *Cancer* **77**: 1501–1509

Uchimura M, Waki S, Kida E, Kanda K, Narita K, Maeda S, Soejima H, Okamoto K and Ozawa A (1991) Anomalous arrangement of pancreaticobiliary ductal system and biliary carcinoma. *J Biliary Tract Pancreas* **12**: 397–404

*© 2001 Cancer Research Campaign*
